# Supplementary material for: International Comparison of the Levels and Potential Correlates of Objectively Measured Sedentary Time and Physical Activity among Three-to-Four-Year-Old Children
Source: Int J Environ Res Public Health. 2019 May 31;16(11):1929. doi: 10.3390/ijerph16111929 (PMC6603940; doi:10.3390/ijerph16111929)
Supplement: Supplementary file 1 [file ijerph-16-01929-s001.pdf]

Table 1. Average daily minutes spent in sedentary time, total physical activity, and moderate-to-vigorous physical activity by the different correlates.

Correlate	N	Wear Time		Sedentary Time		Total Physical Activity		Moderate-to-Vigorous Physical Activity	
		Mean	SD	Mean	SD	Mean	SD	Mean	SD
**Overall**	1052	697.27	70.18	490.18	72.33	207.08	51.97	87.33	34.09
Age									
3	343	696.34	72.37	492.10	74.18	204.23	54.24	85.20	35.82
4	709	697.71	69.14	489.25	71.45	208.46	50.82	88.36	33.19
Gender									
Male	528	699.57	70.46	482.89	73.40	216.68	52.36	94.90	35.37
Female	524	694.94	69.89	497.53	70.54	197.41	49.79	79.70	30.96
Country									
UK	426	660.05	54.92	454.21	65.13	205.84	51.58	85.42	32.06
Switzerland	142	703.97	63.24	501.78	69.96	202.19	44.77	80.14	27.22
Belgium	104	688.71	55.08	509.16	55.27	179.54	51.30	65.45	32.07
USA	380	738.82	68.10	520.97	67.41	217.85	52.07	98.13	35.25
Season									
Winter	136	704.25	74.07	507.03	63.18	197.22	53.52	80.96	35.88
Spring	110	727.96	68.44	512.26	66.62	215.70	58.45	90.60	41.96
Summer	117	719.82	57.91	502.55	61.64	217.27	44.52	95.79	29.55
Autumn	689	687.16	69.21	481.22	74.99	205.93	51.37	86.62	32.77
Ethnicity									
White	200	705.28	67.01	500.29	63.97	204.98	50.30	87.20	31.52
Other	219	752.72	69.08	530.27	69.66	222.45	54.12	102.72	37.96
Parental Education									
Up to and including completion of compulsory vocational training	86	744.30	74.22	511.20	71.47	233.10	50.32	105.95	33.17
Any post-compulsory education including vocational training	300	733.59	65.28	520.94	66.48	212.65	51.59	95.19	35.19
Weekday vs Weekend									
Weekday	1052	704.92	70.45	497.50	72.82	207.42	53.34	87.28	34.90
Weekend	626	652.92	86.97	452.40	93.93	200.52	64.36	81.42	39.12
Time of Sunrise									
Before 07:00	433	707.30	66.75	489.83	73.49	217.47	48.18	93.83	30.92
After 07:00	619	690.24	71.70	490.42	71.56	199.82	53.31	82.78	35.46
Time of Sunset									
Before 19:00	548	688.79	68.74	490.55	67.56	198.23	50.00	81.74	32.42
After 19:00	504	706.48	70.64	489.77	77.24	216.71	52.41	93.40	34.84
Hours of Daylight									
Less than 12 hours	589	690.47	70.27	489.79	69.04	200.68	51.72	83.22	33.88
More than 12 hours	463	705.90	69.17	490.67	76.38	215.23	51.20	92.55	33.67

Note: SD: Standard Deviation.

Table S2. Multi-level unadjusted associations between potential correlates and average daily minutes spent in sedentary time, total physical activity, and moderate-to-vigorous physical activity in children aged 3-to-4-years-old.

Correlate (Reference Category)	N	Sedentary Time					Total Physical Activity					Moderate-to-Vigorous Physical Activity				
		β	(95% CI)	*p*	ICC	R^2	β	(95% CI)	*p*	ICC	R^2	β	(95% CI)	*p*	ICC	R^2
**Age (3 years)**	1052	1.20	(-7.65, 10.05)	0.790	0.119	-0.001	6.60	(-0.20, 13.40)	0.057	0.098	0.028	6.42	(2.06, 10.78)	0.004	0.122	0.001
Gender (Male)	1052	14.43	(6.52, 22.35)	<0.001	0.121	-0.004	-19.39	(-25.41, -13.37)	<0.001	0.098	0.058	-15.58	(-19.41, -11.76)	<0.001	0.123	0.039
Country (UK)	1052				0.000	0.890				0.017	0.739				0.009	0.834
Switzerland		47.57	(35.11, 60.04)	<0.001			3.23	(-15.89, 22.35)	0.741			-2.23	(-12.21, 7.75)	0.661		
Belgium		54.95	(40.88, 69.02)	<0.001			-19.42	(-39.19, 0.35)	0.054			-16.92	(-27.41, -6.43)	0.002		
USA		66.76	(57.68, 75.83)	<0.001			21.57	(4.80, 38.33)	0.012			15.69	(7.05, 24.34)	<0.001		
Season (Winter)	1052				0.133	-0.118				0.123	-0.208				0.120	0.018
Spring		-3.18	(-20.40, 14.04)	0.717			19.26	(6.07, 32.44)	0.004			10.73	(2.24, 19.23)	0.013		
Summer		-10.76	(-28.73, 7.20)	0.240			10.76	(-2.99, 24.51)	0.125			10.96	(2.10, 19.82)	0.015		
Autumn		5.19	(-9.46, 19.85)	0.487			-1.30	(-12.51, 9.92)	0.821			3.16	(-4.06, 10.38)	0.391		
Ethnicity (White)	419	22.86	(9.39, 36.33)	0.001	0.058	0.356	14.03	(3.47, 24.58)	0.009	0.126	0.099	12.18	(5.12, 19.23)	0.001	0.094	0.247
Parental Education (Up to/including compulsory education)	386	8.96	(-7.18, 25.10)	0.277	0.019	0.032	-20.45	(-32.72, -8.18)	0.001	0.000	0.027	-10.76	(-19.07, -2.45)	0.011	0.000	0.016
Weekday vs Weekend (Weekday)	1678	-32.75	(-40.85, -24.65)	<0.001	0.088	0.180	-3.46	(-9.42, 2.49)	0.254	0.109	0.013	-1.53	(-5.25, 2.19)	0.421	0.120	0.016
Time of Sunrise (Before 07:00)	1052	14.69	(6.04, 23.35)	0.001	0.135	-0.127	-14.19	(-20.81, -7.57)	<0.001	0.068	0.312	-7.79	(-12.06, -3.51)	<0.001	0.097	0.201
Time of Sunset (Before 19:00)	1052	-10.02	(-19.10, -0.94)	0.031	0.128	-0.072	18.47	(11.58, 25.35)	<0.001	0.086	0.160	11.55	(7.10, 15.99)	<0.001	0.109	0.123
Hours of Daylight (Less than 12 hours)	1052	-8.89	(-18.07, 0.28)	0.057	0.126	-0.060	14.76	(7.76, 21.75)	<0.001	0.090	0.110	9.72	(5.21, 14.22)	<0.001	0.113	0.080

Note: CI: Confidence Interval, ICC: Intraclass Correlation Coefficient, All models are adjusted for study clustering effects.
